# The Class I Hdac Inhibitor Mgcd0103 Induces Cell Cycle Arrest and Apoptosis in Colon Cancer Initiating Cells by Upregulating *Dickkopf-1* and Non-Canonical *Wnt* Signaling

**DOI:** 10.18632/oncotarget.194

**Published:** 2010-11-19

**Authors:** Shaheen Sikandar, Diana Dizon, Xiling Shen, Zuomei Li, Jeffery Besterman, Steven M. Lipkin

**Affiliations:** ^1^ Department of Medicine, University of California, Irvine, USA; ^2^ Department of Biomedical Engineering, Cornell University, USA; ^3^ Methylgene, Quebec, Canada; ^4^ Departments of Medicine and Genetic Medicine, Weill Cornell Medical College, USA

**Keywords:** Colon cancer, HDAC inhibitors, non-cannonical WNT signaling

## Abstract

Colorectal cancer metastatic recurrence and chemoresistance are major causes of morbidity and mortality. Colon cancer initiating cells (CCIC) are thought to contribute to both these processes. To identify drugs with anti-CCIC activity we screened a number of FDA approved and investigational compounds. We found that the class I selective histone deacetylase inhibitor (HDACi) MGCD0103 has significant activity against CCIC, and also significantly inhibits non-CCIC CRC cell xenograft formation. Both MGCD0103 and the pan-HDAC inhibitor Trichostatin impairs CCIC clonogenicity and cause cell cycle arrest and cell death. Gene expression profiling revealed that the canonical WNT ligand *DKK-1* is a highly upregulated target of HDAC inhibitors. Despite the presence of APC mutations and constitutive WNT signaling in CCIC, both transfected and recombinant *DKK-1* dramatically inhibit CCIC proliferation and clonogenicity. Overall, these data show that inhibition of class I HDACs is a promising novel approach to target both CCIC and non-CCIC CRC cells. Our studies also provide novel insights into roles for DKK1 in addition to canonical WNT signaling and the mechanism of CCIC tumor formation.

## INTRODUCTION

Colorectal cancer (CRC) is the 2^nd^ leading cause of cancer death in the United States. Chemotherapy is the primary form of treatment once CRC has spread beyond the colon. However, in most cases tumors recur and become refractory to chemotherapy. In part, tumor recurrence and chemoresistance is attributed to stochastic genetic and epigenetic changes, which cause selection of resistant clones that form new tumors. Recently, an additional mechanism of tumor recurrence and chemoresistance has been proposed. This mechanism postulates that a minority of cells in a tumor (referred to as cancer stem cells or cancer initiating cells) are intrinsically more chemo- and radiation resistant [1,2]. In support of this model, recent studies have demonstrated that these cells express high levels of DNA damage response genes, which contribute to chemoresistance [3]. They also have lower reactive oxygen species (ROS) levels as another mechanism conferring resistance to radiation, and in fact have less DNA damage after ionizing radiation [4]. These cells also express high levels of drug efflux transporter genes that also lead to chemoresistance and relapse [5].

Recent CRC focused studies show that colon cancer initiating cells (CCIC) are enriched in xenogenic tumors growing in mice treated with chemotherapy, and that CCIC rapidly regenerate new tumors even with concurrent therapy [6,7]. Additionally, high NOTCH signaling levels cause CRC chemoresistance [8] and our own recent studies show that CCIC have 10–30X higher NOTCH levels than non-CCIC CRC cells as a potential mechanism of chemoresistance and tumor recurrence [9].

As CCIC are thought to both self-renew and give rise to non-CCIC CRC cancer cells that populate tumors, epigenetic control of gene expression has been proposed as a likely mechanism to regulate the CCIC to non-CCIC CRC cancer cell transition. Drugs that modulate epigenetic state are therefore a promising approach for anti-CCIC targeted therapy. The covalent modification of histones (such as methylation or acetylation) is an important mechanism of epigenetic regulation. Transcriptionally active gene promoters generally have hyperacetylated chromatin while transcriptionally silent genes have hypoacetylated chromatin. Histone acetyltransferases (HATs) catalyze the addition of acetyl groups onto histones and act as transcription co-activators. Conversely, Histone deacetylases (HDACs) are transcription co-repressors that remove acetyl groups from histones. There are three distinct classes of HDACs. Class I includes HDACs 1,2,3 and 8, Class II HDACs 4-7 and 9-11, and Class III the SIR2 family. HDACs inhibit the expression of target genes to which they are bound by deacetylating Histone 3 at lysines K9 and K14 in target promoters. HDAC inhibitors directly relieve repression of these targets by preventing Histone 3 K9 and K14 de-acetylation. H3 K9/K14 deacetylation causes subsequent trimethylation of H3 on lysine 4 (H3K4me3) to maintain longer-term gene upregulation.

Normal colon mucosa has high levels of Class I HDACs and CRCs have higher histone acetylation levels than normal colon [10,11]. HDAC inhibitor treated *APC* mutant mice develop fewer intestinal adenomas [12]. Therefore, HDAC inhibition, and Class I HDAC inhibition in particular, is thought to be a promising strategy to improve anti-CRC chemotherapy. MGCD0103 is the first Class I selective HDACi to enter clinical trials. Phase I/II clinical studies show that MGCD0103 is active against lymphomas [13-15]. Currently, non-class specific HDACi are FDA approved for treatment of lymphomas. Both class specific and pan-HDACi are also actively being evaluated in the treatment of a variety of solid tumors as well.

*WNT* signalling plays a critical role in both CCIC and non-CCIC CRC cell proliferation and the majority of CRC tumors have increased *WNT* signaling [16,17]. Canonical *WNT* signaling is initiated by ligand binding to Frizzled-Lrp5/6 cell surface receptors. This binding triggers a signaling cascade that causes β-catenin nuclear translocation. β-catenin binds to LEF/TCF transcription factors and upregulates genes important in proliferation and anti-apoptosis, such as MYC and CCD1. *APC* is a core component of the cytoplasmic “destruction complex” that degrades β-catenin via the proteasome. *APC* mutations are very common in CRC and cause constitutive *WNT* signaling by nuclear β-catenin.

Dickopf (DKK) family proteins are extracellular *WNT* antagonists that bind to LRP5/6 with co-factors. *DKK-1* is thought to be the most important family member in CRC. *DKK-1* causes LRP 5/6 endocytosis and downregulation, inhibiting downstream canonical *WNT* signaling [18]. In transgenic mice, targeted overexpression of *DKK-1* to the intestine inhibits proliferation of intestinal epithelial cells in villi and crypts [19]. *DKK-1* also inhibits epithelial cell polarization and migration, processes that are important in tumor progression and metastasis [20].

*DKK-1* expression is downregulated in human CRC. In many tumors *DKK-1* is epigenetically silenced. In colon cancer cell lines where *DKK-1* is epigenetically silenced, forced expression of *DKK-1* inhibits proliferation and reduces xenograft tumor growth. Overall, *DKK-1* is thought to act as a growth suppressor for CRC [21]. However, the mechanism of *DKK-1* growth inhibition is poorly characterized.

We previously derived CCIC from primary CRCs [9]. To understand the mechanism of CCIC tumor formation we screened a variety of drugs for CCIC anti-proliferative activity. These included standard conventional cytotoxic chemotherapy drugs such as 5-FU and oxaliplatin, EGF Receptor inhibitors, *IGF1* Receptor inhibitors, nitrosylated NSAIDs, and targeted agents including sunitinib and sorafenib, among others. CCIC were also resistant to almost all the agents screened, with the exception of the Class I HDACi MGCD0103. MGCD0103 effectively inhibits CCIC proliferation and clonogenicity. Furthermore, MGCD0103 is also active against commonly used non-CCIC CRC cell lines. These data were confirmed with the non-class specific HDACi Trichostatin (TSA). Gene expression profiling revealed that a mechanism of HDACi induced CCIC growth arrest and apoptosis is upregulation of the *WNT* antagonist *DKK-1*. Overall, our data show that MGCD0103 is a promising approach to target CCIC as well as non-CCIC CRC cells. This dual activity is important because CCIC are highly chemoresistant. Our data also provide evidence that *DKK-1* can inhibit proliferation and clonogenicity even in CCIC that carry *APC* mutations. This result is consistent with a role for DKK1 to inhibit CCIC growth through mechanisms in addition to its role in canonical *WNT* signaling pathways and provides insight into the mechanisms of CCIC proliferation, tumor formation and chemoresistance.

## RESULTS

### HDAC inhibitors have anti-CCIC and non-CCIC CRC cell anti-proliferative activity

To test if HDAC inhibitors have anti-tumor capacity in colon cancer we tested if Class I HDAC inhibitor MGCD0103 and TSA affected proliferation in colon cancer cell lines. We found that MGCD0103 had anti-proliferative activity against colon cancer cell lines in MTT assays with an IC50 value of 0.7–1.0μM in commonly used CRC cell lines HCT15, HT-29, SW48 and SW620. For comparison the IC50 value for HMEC cells is 19μM (Table 1). In addition, cell cycle analysis of HCT15 and HCT116 cells treated with MGCD0103 show G2/S phase cell cycle arrest and a sub-G1 accumulation (data not shown). Thus, Class I HDAC inhibitor MGCD0103 inhibits proliferation of colon cancer cell lines and causes cell cycle arrest and apoptosis.

**Table 1:** MTT IC_50_ Values (mM) of MGCD0103 in Different Human Cancer and Normal Cell Lines

CELL LINE	IC 50 (μM)
HCT15	0.7
HT-29	0.7
SW48	0.8
SW620	1.0
HMEC	19

**Figure 1:**
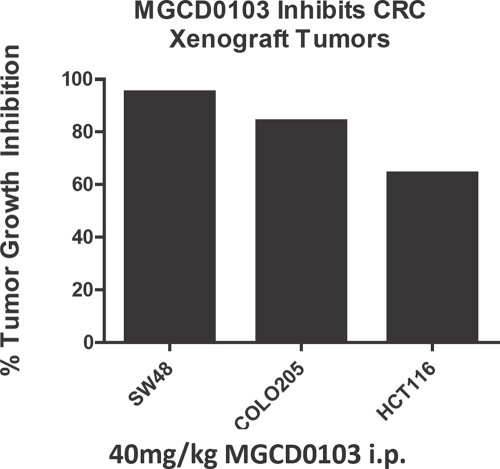
MCCD0103 inhibits xenograft growth of multiple commonly used CRC cell lines NOD/SCID mice were injected in the lateral flank with one million cells of the indicated cell line and mice were treated with 40mg/kg MGCD0103 i.p. daily injections for three weeks. The decrease in xenograft tumor volume is indicated. Inhibition is the mean of two independent experiments for each cell line.

To test if MGCD0103 had anti-CRC activity in xenograft models we treated mice injected with commonly used CRC cell lines including SW48, Colo 205 and HCT116. When the tumors reached ~100mm^3^ mice were randomized into groups of 6–8 animals and treated with MGCD0103 i.p. or vehicle. Tumors were measured 2–3 times a week for up to 3 weeks. Treatment of mice with MGCD0103 had anti-tumor activity in all commonly used CRC cell lines tested, as well as other solid tumor cell lines. For colon cancer the growth inhibition was 60% in an aggressive xenograft model such as HCT116 and almost complete growth inhibition in Colo205 (Figure 1).

### MGCD0103 inhibits CCIC viability

**Figure 2:**
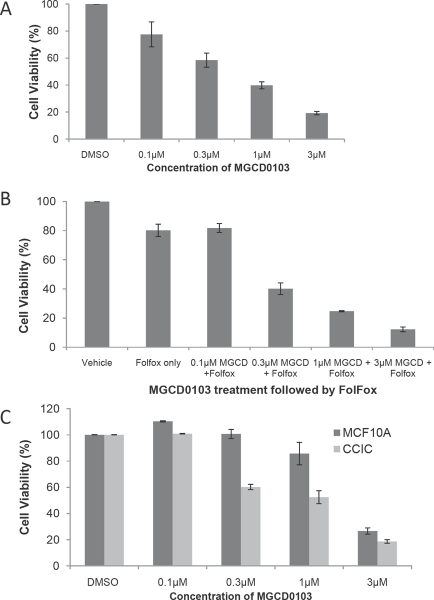
MGCD0103 impairs viability of CCIC Cells were cultured in stem cell media in suspension and treated with MGCD0103 and/or the combination of 5-FU/Oxaliplatin (FOLFOX). MTT assays were performed to assess cell viability after drug treatment. Error bars are S.E.M.

After demonstrating that MGCD0103 has anti-tumor activity in non-CCIC CRC cell lines, we next evaluated anti-CCIC activity, comparing MGCD0103 to standard conventional cytotoxic chemotherapy agents such as 5-FU, and oxaliplatin and SN38/CPT-11. CCIC viability was significantly impaired by MGCD0103 (Figure 2A). Consistent with previous results, CCIC are highly resistant to 5FU/oxaliplatin [7] (Figure 2B). Combining 5FU/oxaliplatin and MGCD0103 further decreased CCIC viability and proliferation in a dose dependent manner (Figure 2B). To determine if this effect was specific to CCIC we treated CCIC and normal epithelial cell lines in the same experiment. When treated with MGCD0103, CCIC viability was impaired significantly more than MCF10A cells (Figure 2C). These data show that the same concentration of MGCD0103 reduces CCIC viability more effectively than the other cell types tested. Similar results were obtained when cells were treated with a pan-HDAC inhibitor TSA (data not shown).

### MGCD0103 inhibits CCIC clonogenicity and causes apoptosis in CCIC

**Figure 3:**
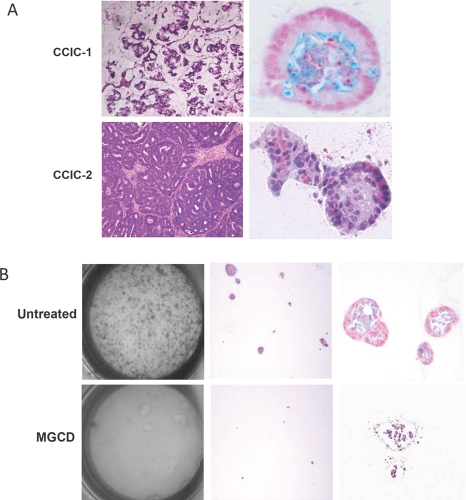
MGCD0103 inhibits CCIC D culture tumor formation **A.** Representative pictures of xenograft tumors (left) and 3D culture glandular crypt-like tumors (right) from CCIC cell lines. **B.** Light microscopy view of 10cm plate of CCIC after MGCD0103 treatment in 3D matrigel culture at low magnification (left), 10X (middle) and 40X (right).

Next we evaluated whether MGCD0103 inhibited the ability of CCIC to form tumour foci *in vitro* we used a 3D matrigel assay. In this assay CCIC are plated as single cells form tumor foci with organized glandular crypt like lumens and give rise to cells that express non-CCIC CRC cell tumor markers [9] (Figure 3A). Using the 3D matrigel *in vitro* culture as previously described we treated CCIC with MGCD0103 for 72h and then cultured in normal media. We then quantified CCIC tumor formation in 3D culture in vitro. MGCD0103 treated cells formed no tumor foci. Only a few single, isolated CCIC cells were still observed (Figure 3B). Morphologically, cells have apoptotic bodies and lose self-renewal (Figure 3B and data not shown). In summary, both MTS and 3D tumor formation assays are consistent with inhibition of proliferation as a mechanism of MGCD0103 action. Similar results were seen with TSA treatment (data not shown). Furthermore, cells treated with MGCD0103 and TSA were cultured in 3D cultures for up to 2 months after treatment to evaluate if cells can recover from a pulse of HDACi Even after two months of culture CCIC failed to recover and form tumor foci in 3D culture as compared to control. This suggests that HDAC inhibitors not only inhibit proliferation but can induce long-term (possibly permanent) changes in the CCIC epigenetic state that inhibit tumor formation.

**Figure 4:**
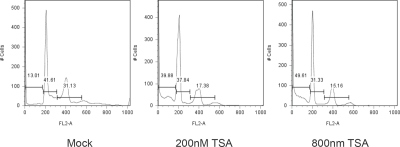
HDAC inhibitors induce CCIC apoptosis FACS cell cycle analysis plot of CCIC treated with vehicle only (mock), 200nM TSA or 800nM TSA for 24 hours.

To understand if HDACi treatment causes CCIC cell death we performed FACS and cell cycle analysis. This revealed that CCIC initiate apoptosis, indicated by the presence of a sub-G1 peak is present in CCIC treated with TSA (Figure 4A). In summary, HDACi causes CCIC cell cycle arrest, which is followed by cell death.

### HDAC inhibitors induce expression of DKK-1

**Figure 5:**
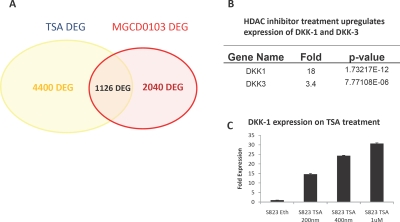
Gene expression profiling reveals CCIC targets of MGCD0103 and TSA upregulation **A.** Venn diagram of differentially expressed genes (DEG) common for 2 CCIC cell lines treated with TSA or MGCD0103. **B.** Treatment with HDAC inhibitors upregulates expression of DKK-1 and DKK-3. Fold upregulation and p-value from quadruplicate data points for each of 2 CCIC lines from Affymetrix array data is shown. **C.** Q-PCR confirmation of DKK-1 upregulation by increasing concentrations of TSA.

The epigenetic state of CCIC is thought to be different from non-CCIC CRC cell lines. To identify the mechanism of HDACi induced growth arrest and apoptosis we performed gene expression profiling of two distinct CCIC lines treated with 0.7 μM MGCD0103, 1 μM TSA or mock control for 6 hours. The short time period after treatment was used in order to focus on direct targets of HDAC inhibition rather than downstream indirect transcriptional effects. We used Cyber T analysis to find differentially expressed genes between both groups *i.e.* control *vs.* MGCD0103 and control *vs.* TSA. The pan-HDAC inhibitor TSA treatment caused differential gene expression (DEG) of 4440 target genes common to both CCIC lines, and the Class I HDAC inhibitor MGCD0103 caused DEG of 2040 genes in the same lines. In many experiments, gene array studies can have a high false-positive rate. To minimize the false positive rate, we focused our analysis on genes regulated up or down that were common to both the pan-HDAC and class I specific HDAC inhibitors and seen in both CCIC lines, which gave a set of 1126 DEG. The significantly regulated genes (PPDE = 0.99) in each group were then overlapped to find a common subset of genes that are differentially expressed in both treatment groups (Figure 5A). The gene list was used in NIH DAVID (Database for Annotation, Visualization and Integrated Discovery) (http://david.abcc.ncifcrf.gov/) resource. DNA damage response and cell cycle arrest were among the top GO categories that are enriched (Table 2). Notably, the expression of the WNT antagonist *DKK-1* increased 18-fold in CCIC treated with TSA and 7.7-fold in MGCD0103 treated CCIC. To validate the array data we performed q-PCR analysis for *DKK-1* on cells treated with increasing concentrations of TSA. TSA induces *DKK-1* expression in a dose dependant manner, thus validating the array data (Figure 5B). Induction of *DKK-1* by MGC0103 is not as robust as TSA under the time frame (6 hr) in qRT-PCR validation (data not shown). Overall, these analyses were consistent with a mechanistic role for *DKK-1* in HDACi induced CCIC growth arrest and apoptosis.

**Table 2:** Top statistically enriched Gene Ontology biological process categories for differentially expressed genes

Term	Count	%	PValue	Benjamini
GO:0007049 cell cycle	66	11.40%	5.84E-15	3.09E-11
GO:0022402c ell cycle processes	52	8.98%	7.76E-11	2.04E-07
GO:0006259~DNA metabolic process	54	9.33%	1.19E-09	2.08E-06
GO:0006334~nucleosome assembly	15	2.59%	4.37E-08	5.74E-05
GO:0000074~regulation of progression through cell cycle	36	6.22%	1.58E-07	1.66E-04
GO:0006950~response to stress	57	9.84%	1.72E-07	1.51E-04
GO:0051726~regulation of cell cycle	36	6.22%	1.80E-07	1.35E-04
GO:0031497~chromatin assembly	15	2.59%	2.53E-07	1.66E-04
GO:0065004~protein-DNA complex assembly	18	3.11%	3.35E-07	1.96E-04
GO:0006270~DNA replication initiation	9	1.55%	7.03E-07	3.69E-04
GO:0006260~DNA replication	22	3.80%	1.43E-06	6.84E-04
GO:0006333~chromatin assembly or disassembly	16	2.76%	3.75E-06	0.001639764
GO:0006974~response to DNA damage stimulus	24	4.15%	7.93E-06	0.00319912
GO:0022403~cell cycle phase	25	4.32%	1.19E-05	0.004469104
GO:0000279~M phase	22	3.80%	1.26E-05	0.004392701
GO:0006268~DNA unwinding during replication	6	1.04%	2.38E-05	0.007792704
GO:0009719~response to endogenous stimulus	26	4.49%	3.25E-05	0.009990679
GO:0032508~DNA duplex unwinding	6	1.04%	4.71E-05	0.013652474
GO:0032392~DNA geometric change	6	1.04%	4.71E-05	0.013652474
GO:0048523~negative regulation of cellular process	51	8.81%	6.61E-05	0.017204735
GO:0048519~negative regulation of biological process	52	8.98%	9.79E-05	0.024198433
GO:0006261~DNA-dependent DNA replication	12	2.07%	1.01E-04	0.023756591
GO:0006325~establishment and/or maintenance of chromatin architecture	21	3.63%	1.45E-04	0.0326071
GO:0006281~DNA repair	19	3.28%	1.48E-04	0.031823146
GO:0000075~cell cycle checkpoint	9	1.55%	1.53E-04	0.031601678
GO:0000278~mitotic cell cycle	21	3.63%	1.65E-04	0.032719024
GO:0006323~DNA packaging	21	3.63%	1.87E-04	0.03579105
GO:0065003~macromolecular complex assembly	30	5.18%	1.95E-04	0.035944691
GO:0007067~mitosis	17	2.94%	2.07E-04	0.036736724
GO:0000087~M phase of mitotic cell cycle	17	2.94%	2.28E-04	0.039230324

### DKK-1 inhibits CCIC proliferation

**Figure 6:**
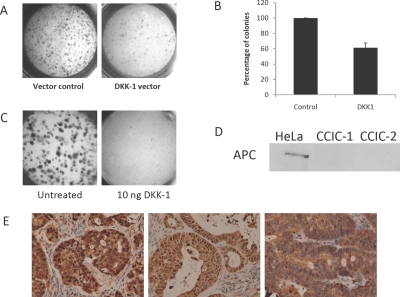
DKK1 inhibits CCIC tumor formation through non-canonical WNT signaling pathways Light microscopy of CCIC 3D culture clonogenicity in cells transfected with DKK-1 expression vector (**A**) or addition of recombinant DKK-1 (**C**). **B.** Reduction in CCIC colony number with DKK-1 transfection. Error bars are S.E.M. **D.** Western blot showing loss of wild-type APC in CCIC-1 and CCIC-2 lines. Hela WT APC is shown as a positive control. **E.** Immunohistochemistry of CCIC xenograft tumors stained for beta-catenin.

To test if *DKK-1* induced CCIC growth arrest and apoptosis we first transfected CCIC with an expression vector for *DKK-1* or GFP control. Equal numbers of CCIC were plated in 3D culture system to assay tumor foci formation. Cells transfected with *DKK-1* had fewer and smaller tumor foci vs. GFP control (Figure 6A,B). Next, we used recombinant *DKK-1* to treat CCIC already plated in 3D assays. Again, *DKK-1* caused fewer and smaller tumor foci vs. control (Figure 6C). *DKK-1* inhibition of *WNT* signaling is upstream of *APC* and the beta-catenin destruction complex. As mutations in *APC* are common in CRC we tested if *APC* is mutated in CCIC. Western analysis revealed that the two CCIC lines studied both have *APC* protein truncations and no WT *APC* protein (Figure 6D). Next, we stained for β-catenin in xenograft samples from these CCIC lines. Nuclear β-catenin is an indicator of active *WNT* signaling. We found that nuclear beta-catenin is present in xenografts derived from both lines and is consistent with active *WNT* signaling (Figure 6E). Similar results were seen with 3Dculture CCIC tumors (data not shown). Overall, our data are consistent with *DKK-1* as a potent inhibitor of CCIC proliferation and tumor formation, but through a mechanism that is independent of canonical *WNT* signaling. DISCUSSION:CRC metastatic recurrence and chemoresistance are major causes of cancer related death in the United States. Recent experiments have implicated a role for CCIC in both of these processes. Identifying novel compounds and drug combinations that target both CCIC and non-CCIC CRC cells is an important approach to improve CRC patient outcomes.

To identify potential anti-CCIC therapeutics we screened through a number of FDA approved and investigational drugs and found the class I HDACi MGCD0103 to be the most effective of the agents tested. We also found MGCD0103 to effectively inhibit the growth of non-CCIC CRC cells. Since Class I HDACs 1-3 are over expressed in CRC [22] this latter finding is not completely unexpected. However, because CCIC are a minority of cells in tumors, the ability of Class I HDACi to inhibit these cells as well as non-CCIC bulk CRC cells is potentially important. Drugs inhibiting both CCIC and non-CCIC CRC cell tumor formation such as MGCD0103 are anticipated to be particularly promising candidates to take forward in CRC developmental therapeutic clinical trials.

MGCD0103 and TSA induce CCIC cell cycle arrest, and apoptosis. Mechanistically, our study provides insights into the primary targets of HDAC inhibition in CCIC. *DKK-1* is epigenetically silenced in many CRCs but can be dramatically up-regulated by treatment with MGCD0103 or TSA. Consistent with a functional role for *DKK-1*, both transfected and recombinant *DKK-1* significantly reduce CCIC proliferation and clonogenicity in 3D cultures. Overall, our results suggest *DKK-1* may be a useful pharmacogenetic biomarker for MGCD0103 clinical trials for CRC and possibly other solid tumors. Previous studies have shown that promoter hypermethylation causes *DKK-1* silencing in CRC and that this is not an early event but more closely associated with late tumor progression [21]. Because epigenetic state is thought to play an important role in the CCIC to non-CCIC CRC cell transition, it is tempting to speculate that HDACi upregulation of *DKK-1* transcription in CCIC may prevent subsequent promoter methylation in non-CCIC CRC daughter cells. Future immunohistochemistry co-localization studies in CRCs of *DKK-1* and CCIC markers such as ALDH1 or CD44/CD166 (and in parallel co-localization studies with non-CCIC CRC cell markers such as CEA) will be useful to understand the precise role of *DKK-1* expression in both CCIC and non-CCIC CRC cells.

Constitutive activation of canonical *WNT* signaling is a common feature of nearly all CRCs and *DKK-1* has a clearly established role as a canonical *WNT* pathway inhibitor. Mutations in *APC* and less frequently *β-CATENIN* and *AXIN* are known to cause constitutive downstream signaling independent of upstream signals. However recent reports have suggested that upstream signaling from *WNT* inhibitors such as SFRP1, WIF-1 and *DKK-1* inhibit CRC cell growth even in presence of downstream mutations [21]. In contrast to sFRPs which decrease levels of β-CATENIN/LEF1 dependant transcription even in cells carrying *APC* mutations, *DKK-1* has minimal effect on these targets [21]. Because the CCIC lines we studied do not express WT *APC*, our study provides new evidence that *DKK-1* inhibits proliferation through mechanisms that are independent of canonical *WNT* signaling. In mesothelioma cells *DKK-1* activates the JNK pathway to induce apoptosis [23]. Since JNK signaling increases intestinal tumorgenesis in mouse models [24], future additional studies will be helpful to understand the role of JNK and other anti-proliferative mechanisms of *DKK-1* in CCIC and non-CCIC CRC cells that are independent of canonical *WNT* signaling.

## MATERIALS AND METHODS

### MGCD0103 and TSA treatment of CCIC

CCIC were plated in 3D culture (DAY 0) and treated with 0.7μM MGCD0103 (DAY 1) or 200nM TSA and treated for 48h. Controls were treated with DMSO or ethanol respectively. The cells were then cultured without drug for another 14 days or upto 2 months to assay number of colonies formed.

### CCIC transfection and DKK-1 treatment

CCIC were transfected with *DKK-1* expression vector (a kind gift from Dr. Marian Waterman) or GFP expression vector using Lipofectamine 2000 overnight. The cells were then trypsinzed counted and equal number of live cells were plated in each well for 3D assays. Recombinant *DKK-1* was purchased from R&D systems. CCIC were plated in 3D wells and treated with rec*DKK-1* at 10ng. CCIC were cultured for 14 days to assay affect on proliferation and colony formation.

### Protein Isolation and Western Blotting

CCIC spheres were treated with TSA and cells were collected at different time points. The cell pellets were lysed in ice cold NP40 lysis buffer (50mM Tris-HCl, pH7.5, 150mM NaCl, 1mM EDTA and 1% NP-40). Protein quantification was carried out using Bio-Rad Bradford protein quantification assay. Proteins were separated by SDS/PAGE and transferred to Immobilon-P PVDF (Millipore). Membranes were blocked with 5% nonfat dry milk and then incubated overnight at 4 °C with specific primary antibodies for Acetyl H3K9 and Acetyl H3K4 (Upstate Biotech) at dilutions recommended by the manufacturer. Detection was carried out by peroxidase based chemiluminesence and Dura West substrate (Pierce).

### Gene expression profiling

Two CCIC lines were treated with MGCD0103 (0.71 μM) or TSA (200 nM) for 6h in quadruplicates and RNA was extracted using Qiagen RNeasy Spin columns. Expression profiling was carried out using total RNA from CCIC lines on Affymetrix GeneChip Human Exon 1.0 ST Array. RNA extraction, labeling, hybridization and scanning were carried out according to recommended protocols. The Cybet-T program was used to determine statistically significant differentially expressed genes compared to mock treated samples. Differentially regulated genes were analyzed by Ingenuity Pathway Analysis.

### RNA isolation and quantitative RT-PCR

Total RNA was extracted using Qiagen RNeasy Spin columns and reverse transcribed using ABI High capacity cDNA kit. Taqman Assay on demand was used to quantify gene expression on a ABI PRISM HT7900. Gene expression was normalized using GAPDH or RPLPO.

## References

[1] Cho R.W., Clarke M.F. (2008). Recent advances in cancer stem cells. Curr Opin Genet Dev.

[2] Zhou B.-B.S. (2009). Tumour-initiating cells: challenges and opportunities for anticancer drug discovery. Nat Rev Drug Discov.

[3] Bao S. (2006). Glioma stem cells promote radioresistance by preferential activation of the DNA damage response. Nature.

[4] Diehn M. (2009). Association of reactive oxygen species levels and radioresistance in cancer stem cells. Nature.

[5] Hirschmann-Jax C. (2004). A distinct “side population”of cells with high drug efflux capacity in human tumor cells. Proceedings of the National Academy of Sciences of the United States of America.

[6] Dylla S.J. (2008). Colorectal cancer stem cells are enriched in xenogeneic tumors following chemotherapy. PLoS ONE.

[7] Todaro M. (2007). Colon cancer stem cells dictate tumor growth and resist cell death by production of interleukin-4. Cell Stem Cell.

[8] Meng R.D. (2009). gamma-Secretase inhibitors abrogate oxaliplatin-induced activation of the Notch-1 signaling pathway in colon cancer cells resulting in enhanced chemosensitivity. Cancer Res.

[9] Sikandar S.S. NOTCH Signaling Is Required for Formation and Self-Renewal of Tumor-Initiating Cells and for Repression of Secretory Cell Differentiation in Colon Cancer. Cancer Research.

[10] Fraga M.F. (2005). Loss of acetylation at Lys16 and trimethylation at Lys20 of histone H4 is a common hallmark of human cancer. Nat Genet.

[11] Ishihama K. (2007). Expression of HDAC1 and CBP/p300 in human colorectal carcinomas. J Clin Pathol.

[12] Zhu P. (2004). Induction of HDAC2 expression upon loss of APC in colorectal tumorigenesis. Cancer Cell.

[13] Bonfils C, Kalita Ann, Liu J, Besterman JM, Li Z (2005). Development of Whole Cell HDAC Enzyme Assay to Analyze Inhibitory Activity of MGCD0103 In Vitro and In Vivo. 96th AACR Annual Meeting.

[14] Li Z, Zhou Nancy Z, Fournel M, Rahil G, Wang J, Delorme D, Moradei O, MacLeod R, Besterman J (2004). Antitumor Activities of MGCD0103, a Novel and Isotype-Selective Histone Deacetylase Inhibitor. 16th EORTC-NCI-AACR (Symposium on Molecular Targets and Cancer Therapeutics).

[16] Kinzler K.W., Vogelstein B. (1996). Lessons from Hereditary Colorectal Cancer. Cell.

[17] Vermeulen L. (2010). Wnt activity defines colon cancer stem cells and is regulated by the microenvironment. Nat Cell Biol.

[18] Bafico A. (2001). Novel mechanism of Wnt signalling inhibition mediated by Dickkopf-1 interaction with LRP6/Arrow. Nat Cell Biol.

[19] Kuhnert F. (2004). Essential requirement for Wnt signaling in proliferation of adult small intestine and colon revealed by adenoviral expression of Dickkopf-1. Proceedings of the National Academy of Sciences of the United States of America.

[20] Koch S. (2009). Dkk-1 Inhibits Intestinal Epithelial Cell Migration by Attenuating Directional Polarization of Leading Edge Cells. Mol. Biol. Cell.

[21] Aguilera O. (2006). Epigenetic inactivation of the Wnt antagonist DICKKOPF-1 (DKK-1) gene in human colorectal cancer. Oncogene.

[22] Weichert W. (2008). Class I Histone Deacetylase Expression Has Independent Prognostic Impact in Human Colorectal Cancer: Specific Role of Class I Histone Deacetylases In vitro and In vivo. Clinical Cancer Research.

[23] Lee A.Y. (2004). Dickkopf-1 antagonizes Wnt signaling independent of [beta]-catenin in human mesothelioma. Biochemical and Biophysical Research Communications.

[24] Sancho R. F-box and WD Repeat Domain-Containing 7 Regulates Intestinal Cell Lineage Commitment and Is a Haploinsufficient Suppressor of Intestinal Tumorigenesis. Gastroenterology.

